# Compressing DNA sequence databases with coil

**DOI:** 10.1186/1471-2105-9-242

**Published:** 2008-05-20

**Authors:** W Timothy J White, Michael D Hendy

**Affiliations:** 1 Allan Wilson Centre for Molecular Ecology and Evolution, Massey University, Palmerston North, New Zealand

## Abstract

**Background:**

Publicly available DNA sequence databases such as GenBank are large, and are growing at an exponential rate. The sheer volume of data being dealt with presents serious storage and data communications problems. Currently, sequence data is usually kept in large "flat files," which are then compressed using standard Lempel-Ziv (gzip) compression – an approach which rarely achieves good compression ratios. While much research has been done on compressing individual DNA sequences, surprisingly little has focused on the compression of entire databases of such sequences. In this study we introduce the sequence database compression software coil.

**Results:**

We have designed and implemented a portable software package, coil, for compressing and decompressing DNA sequence databases based on the idea of *edit-tree coding*. coil is geared towards achieving high compression ratios at the expense of execution time and memory usage during compression – the compression time represents a "one-off investment" whose cost is quickly amortised if the resulting compressed file is transmitted many times. Decompression requires little memory and is extremely fast. We demonstrate a 5% improvement in compression ratio over state-of-the-art general-purpose compression tools for a large GenBank database file containing Expressed Sequence Tag (EST) data. Finally, coil can efficiently encode incremental additions to a sequence database.

**Conclusion:**

coil presents a compelling alternative to conventional compression of flat files for the storage and distribution of DNA sequence databases having a narrow distribution of sequence lengths, such as EST data. Increasing compression levels for databases having a wide distribution of sequence lengths is a direction for future work.

## Background

The advent of the Sanger sequencing method enabled DNA sequence data to be collected and manipulated on computers, paving the way for explosive growth in the new field of bioinformatics. Publicly available DNA sequence databases such as GenBank play a crucial role in collecting and disseminating the raw data needed by researchers in the field. This database currently contains 168 Gb of sequence data [1] section 2.2.8, and is expected to continue to grow at an exponential rate, doubling in size roughly every 14 months [2]. The volume of data being dealt with now presents serious storage and data communications problems. Currently, sequence data is usually kept in large "flat files," which are then compressed using standard Lempel-Ziv compression [3] (e.g. with gzip [4]). Unfortunately this approach rarely achieves good compression ratios: typically, gzip fails to match the "compression" afforded by simply encoding each base using 2 bits [5].

Previous work concerning the compression of biological (DNA or protein) sequences can be divided into two categories: techniques developed for efficiently compressing sequence data for the sake of reduced resource consumption (disk space or network usage) [5-9]; and investigations of the usefulness of compressibility as a measure of information content, for the purpose of making inferences about sequences (such as the relatedness of two sequences) [10,11]. In this article we will focus on work in the former category. Examining this body of work reveals two distinct approaches:

• Compressing individual biological sequences

• Compressing databases of biological sequences

### Compressing individual biological sequences

It is now widely recognised that DNA data is inherently difficult to compress below the level of 2 bits per base achievable through direct encoding [5,6,9]. Much research has gone into developing algorithms for more effectively compressing individual DNA sequences. These include BioCompress [6], BioCompress-2 [7], GenCompress [8], the CTW+LZ algorithm [5], and DNACompress [9]. Perhaps the best of these is DNACompress, which employs the PatternHunter [12] sequence search algorithm to discover patterns of approximate repeats or approximate palindromic repeats in sequence data. DNACompress achieved compression averaging 13.7% on a sample set of DNA sequences and is substantially faster than earlier algorithms. Grumbach and Tahi [7] allude to a "vertical" mode of compression for compressing multiple sequences in a database, however they do not elaborate on how this might be accomplished.

While these single-sequence algorithms are interesting from a theoretical point of view, and are certainly becoming increasingly practical in the modern world of genome-scale analysis, a great deal of everyday bioinformatics work continues to entail the communication and storage of multiple sequences, and the modest compression gains afforded by these algorithms are ultimately not sufficient to justify their adoption for large databases.

### Compressing databases of biological sequences

Strelets and Lim [13] describe a program, SAGITTARIUS, for compressing PIR-format [14] protein sequence databases. Their system uses standard dictionary-style compression of sequence entry metadata, and a novel alignment-based compression strategy for the protein sequence data itself. A small number of sequences is maintained in memory as the *reference sequence accumulator*, and each sequence in the database is aligned with each sequence in this list. If any alignment produces a strong match, the input sequence is recoded using symbols describing insertions and deletions to enable recovery from its close match in the accumulator; otherwise, the sequence is output verbatim and added to the accumulator, overwriting the oldest incumbent sequence if the accumulator is full. Sequences to be output are compressed using run-length encoding and Huffman encoding, and the shorter of the two encodings is chosen. Thus the accumulator represents a window of recently encountered interesting sequences. The authors set the size of the accumulator at three sequences, and were able to achieve 2.50:1 compression, significantly better than PKZIP^© ^[15] at 2.13:1.

Strelets and Lim [13] were interested in producing a compressed database that could be used interactively in much the same way as the original database. This was facilitated in part by the fact that their approach never requires recursive decoding of sequences – each sequence is encoded in terms of at most one other sequence, which is itself available "as-is," (i.e. not compressed in terms of another sequence). While useful for interactive operations, it is clear that avoiding recursive encoding must limit the overall level of compression obtained. Since we are targeting maximum compression, coil differs from that of [13] in this respect. Another difficulty arises in the assumption that similar sequences are likely to appear near each other in the input file. This is crucial in order to be able to limit the size of the accumulator and thereby the runtime. The authors found that increasing the size of the accumulator past three sequences increased the runtime but made no substantial improvement in compression, which appeared to justify their assumption. Unfortunately, while this neat localisation of similar sequences may have been true of the PIR database in 1995, it is certainly not true of the large nucleotide databases of today, and we chose not to make this assumption.

Li, Jaroszewski and Godzik have taken a similar approach to the related problem of producing non-redundant protein databases with their CD-HI [16] and CD-HIT [17] packages. More recently, Li and Godzik have extended this approach to DNA sequences with the cd-hit-est program [18]. Their main advance over [13] is in employing short-word filters to rapidly determine that two sequences cannot be similar, which significantly reduces the number of full alignments necessary. Despite impressive speed on small-to-medium datasets, they report that clustering 6 billion ESTs at 95% similarity takes 139 hours [18].

The program nrdb [19] locates and removes exact duplicate sequences from a DNA database in FASTA format. While this program is clearly a step in the right direction, many sequences in a typical database are almost but not quite exact duplicates of other sequences (perhaps differing at one or two positions), highlighting opportunities for further improvements.

### Compression and the maximum parsimony criterion

A *phylogenetic tree *is a Steiner tree estimating the evolutionary history of a set of *taxa *(species, genes or individuals). *Maximum parsimony *is a criterion for building phylogenetic trees from DNA sequences that aims to identify the tree or trees containing the fewest *point mutations *along their edges, where a point mutation, or *edit*, is an insertion or deletion of a single nucleotide or a substitution of one nucleotide for another. (Most implementations of maximum parsimony search consider only point substitutions, for reasons of computational efficiency.) We then note:

*If we are given the complete sequence at one node of a tree, as well as all edge mutations, we can reconstruct the sequences at all the nodes*.

**Figure 1:**
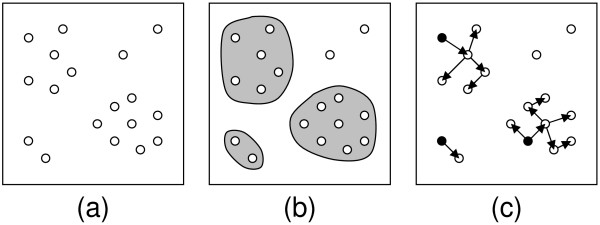
**Edit-tree coding of similar sequence groups**. Circles represent DNA sequences in a database; the straight-line distance between circles represents the edit distance between sequences. Initially (a) we are presented with the input database. In the first step (b), groups of similar sequences are discovered. In the second step (c), each group is edit-tree coded independently by determining a reasonable tree, selecting a root sequence (coloured black) and recording the necessary edits along each edge. Some sequences are not sufficiently similar to any other sequence to be delta-encoded – these sequences will be recorded verbatim.

Thus a maximum parsimony tree represents an optimal solution to storing sequence data in the form of a list of edit operations on a tree rooted at a single reference sequence. This can be an efficient compression if the sequences are closely related, so that the number of edit operations is small in comparison with the total sequence length. Within a large database, it is expected that there will be large groups of closely related sequences – for example, the DNA encoding a particular gene from many different species. More precisely, we expect that many sequences will be highly similar to at least one other sequence in the database. If this is the case a considerable saving in storage space can be achieved by identifying such groups, determining good trees for them, and encoding each group as a single root sequence plus a series of "deltas" along the tree edges. We have called this approach *edit-tree coding*. Figure 1 illustrates how sequences within a database are processed according to this scheme.

In practice, it soon becomes apparent that even a heuristic maximum parsimony search on subsets of sequences is not computationally feasible for a large database. The standard maximum parsimony tree evaluation algorithm requires all sequences to be *aligned*. Both alignment and the subsequent parsimony searches are hard problems [20,21]. Fortunately, when dealing with data compression we are not concerned about exactly maximising some function – our requirement is a method which is fast and performs well on typical cases. A practical alternative to maximum parsimony search is to construct an approximation to the *minimum spanning tree *on the sequences, where the metric is the *edit distance *between two sequences – the number of single-character insertions, deletions or replacements required to transform one sequence into the other. Unlike Steiner trees, minimum spanning trees do not introduce new internal vertices, and computation is fast: an algorithm having time complexity almost linear in the number of edges exists [22]. The total tree length is bounded by twice that of the maximum parsimony tree. By judicious selection of algorithms and data structures, we have developed heuristics and approximations that make this task feasible for databases having sizes in the gigabyte range, despite having essentially quadratic time complexity in the size of the database.

### Goals of coil

Our goal was to develop a software package, coil, for compressing and decompressing DNA sequence databases based on edit-tree coding. The primary intention is to reduce the bandwidth required to transmit large amounts of DNA sequence data from a central repository to many recipients, and also to reduce disk space requirements for archival storage of such data. While it is desirable to enable efficient searching of a compressed database, and progress has been made in this area [23-25], we have not attempted to do so here. Instead, coil is geared towards maximising compression ratios. This is achieved at the expense of execution time and memory usage – but note that the compression time represents a "one-off investment" whose cost is quickly amortised if the resulting compressed file is transmitted many times. Decompression requires little memory and takes O(*D*) time for data sets of size *D *nucleotides.

coil primarily targets sequence databases containing many short sequences of roughly equal length, such as Expressed Sequence Tag (EST) databases. Many such databases exist: at the time of printing, GenBank contains 23 Gb of EST sequence data in FASTA format, comprising 43,380,458 sequences in total [1]. Targeting these databases simplifies design and implementation: many operations on pairs of sequences take time quadratic in the length of their operands, so shorter sequences are important for high performance; also, assuming reasonably short sequences means that little attention need be paid to the intra-sequence positions of regions of similarity between two sequences.

coil reads and write FASTA format databases [26]. This simple format is easy to work with manually, easy to program input and output routines for, and widely used. Additionally, the format contains a minimum amount of additional context information for each sequence, which allows us to focus on compressing the sequence data.

## Implementation

coil consists of a small group of C programs that perform the compression and decompression steps described below, as well as a Perl script which simplifies the compression process by automating sequences of steps and providing sensible defaults where helpful. Use of these programs is described later.

### Overview

Hereafter, unless otherwise qualified, *D *denotes the number of nucleotides (characters) in a database, *N *the number of sequences, and *L *= *D*/*N *the average length of a sequence. As in the C language, the notation *a *% *b *is used to indicate taking the remainder of *a *modulo *b*, for some non-negative integer *a *and positive integer *b*.

Conceptually, the process of compressing a database with coil proceeds through the following stages:

1. Creating a *similarity graph *that pairs sequences of high similarity. The similarity graph is an edge-weighted undirected graph in which vertices represent sequences and edges exist between highly similar sequences, with edge weights indicating similarity strength.

2. Extracting an *encoding graph *from the similarity graph. The encoding graph is a set of rooted directed trees (formally, arborescences) whose arcs correspond to a subset of the edges in the similarity graph.

3. Encoding each tree in the encoding graph. For each tree, the root sequence is stored verbatim (*raw-encoded*); an in-order traversal is then used to *delta-encode *each other sequence in the tree in terms of its parent sequence.

4. A multi-platform general-purpose compression program, such as gzip or bzip2 [27], is applied to extract further compression gains.

A typical usage pattern in a Unix-like environment would be to use the tar archive program to collect the files of step 3 together, and pipe the resulting file through bzip2 -9 (the -9 command-line option requests maximum compression).

Decompression of a coil archive amounts to inverting the delta-encoding of the final compression stage: for each encoded tree, the root sequence is written out, following which an in-order traversal recovers every other sequence using the (already recovered) parent sequence and the encoded delta information. This takes place after the general-purpose compression step is undone. Note that in general, the *order *of sequences in the recovered FASTA file will be different than in the original FASTA file; if this is undesirable, program options can be set to restore the original order (i.e. an exact copy will be produced).

All of these steps are explained in more detail below.

### Edit distances and similarity graphs

A common way to quantify the similarity between two strings *a *and *b *is to compute the *Levenshtein distance*: the smallest number of single-character insertions, deletions and substitutions required to transform a into b. Ideally, we would compute exact distances between every pair of sequences in the database and output a complete graph with perfect similarity information. But since computation of the Levenshtein distance between two strings of length *m *and *n *takes O(*mn*) time [28] in the general case, a database of size *D *containing *N *roughly equal-length sequences would require O(*N*^2^)O(*D*^2^/*N*^2^) = O(*D*^2^) comparisons. When database sizes are in the gigabyte range, quadratic-time algorithms are not viable.

Instead, coil uses a more efficient related similarity measure derived by counting the number of length-*k *substrings, or *k*-tuples, two strings have in common. For small *k*, calculation of *k*-tuple similarity scores can be made very fast by using a *k*-tuple index data structure (described below) to obtain a list of all sequences in the database that contain a given *k*-tuple in constant time.

### The *k*-tuple index

**Figure 2:**
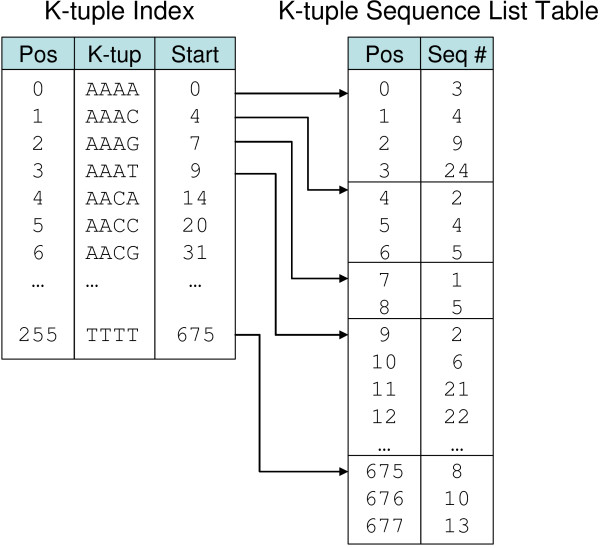
Example *k*-tuple index structure for *k *= 4.

A nucleotide (A, C, G or T) can be encoded as a 2-bit integer, and consequently a *k*-tuple of nucleotides has a natural representation as an integer of 2*k *bits. In coil, the leftmost nucleotide occupies the most significant bits. The *k*-tuple index data structure, which is prepared in a preprocessing step using the program make_index, consists of two files: a k-*tuple sequence list file *ending with the extension .ktl, which contains (*D *- *N*(*k *- 1))/*s *integer sequence numbers (*seqnums*); and a k-*tuple index file *ending with the extension .kti, which contains 4^*k *^integer offsets into the first file. *s *is a "slide" parameter used to reduce the size of the *k*-tuple sequence list file, at the cost of reduced accuracy: only *k*-tuples beginning at sequence positions divisible by *s *are entered into the index. As Figure 2 shows, the *i*th entry in the *k*-tuple index file points to the beginning of the list of seqnums that contain *k*-tuple *i*, which continues until the seqnum list for the (*i *+ 1)th entry begins.

These files are built in O(*D *+ 4^*k*^) time using a bucket sort algorithm that performs two passes over the raw sequence data. The algorithm is similar to that used to build the *k*-tuple indices used by SSAHA [29]. Note that unlike in SSAHA, we do not record the intra-sequence positions of *k*-tuples in the *k*-tuple index, nor do we ever record a given seqnum more than once in a given *k*-tuple's seqnum list; instead we rely on our assumption that the database contains short sequences to ensure that there is a low probability of a sequence containing more than one instance of a particular *k*-tuple. Should this not be the case, the efficacy of the algorithm will be reduced, however correctness will not be compromised.

The bucket sort algorithm requires both files to be able to fit in memory simultaneously. If this is not possible, make_index produces multiple pairs of output files: each pair is an index on a segment of the database that will just fit in the amount of memory specified.

It is worth mentioning that empirically, the sizes of seqnum lists in a *k*-tuple index built from DNA sequence data are highly nonuniform, with some *k*-tuples appearing several orders of magnitude more frequently than others. These *k*-tuples cause many spurious hits that slow down the similarity graph construction step. coil follows the smart practice described in [29] of completely eliminating *k*-tuple seqnum lists that exceed a user-specified size: this has the double effect of reducing index file sizes and dramatically improving the selectivity, and hence the speed, of the next stage.

### Creating the similarity graph

Constructing the similarity graph is the main compression bottleneck in coil. The main contribution made by coil is in engineering an algorithm to efficiently compute pairwise approximate sequence similarity scores using a combination of the raw sequence data and the *k*-tuple index, which is implemented in the find_edges program. We first introduce a "naïve" comparison algorithm, and several variants which each proved unsatisfactory.

The naïve algorithm is parameterised by *k*, *s *and *b*. *b *is a small integer which is used to limit the total number of edges in the similarity graph to *bN*; it is necessary to avoid storing O(*N*^2^) edges in the similarity graph. In most testing, *b *was set to 10. The pseudocode for the algorithm follows:

• For each query sequence *q *in the database:

∘ Create an empty linked list of (seqnum, hit count) pairs, *M*.

∘ For each *k*-tuple *t *in *q*:

▪ Look up the list of sequences that contain *t *starting at a position divisible by *s *using the *k*-tuple index.

▪ Merge this list into *M*.

▪ Keep track of the number of times each sequence in *M *has had a *k*-tuple in common with *q*.

∘ For each pair (*i*, *c*) of the *b *pairs having the highest hit counts in *M*:

▪ Create the edge (*q*, *i*) in the similarity graph and assign it weight *c*.

The seqnum lists read from the *k*-tuple index are in seqnum order, and *M *is maintained in this order also. The merge step is the usual list merge, except that whenever pairs having the same seqnum are to be merged, the result is a single pair whose hit count is equal to the sum of the hit counts of the arguments to the comparison. The intuition here is that if two sequences share many *k*-tuples, they are likely to be similar. In fact, it is relatively straightforward to show that if a string of length *n *is at edit distance *d *from another string, then the two strings must share at least *n *- (*d *+ 1)*k *+ 1 *k*-tuples [30]; so we can reasonably expect a correlation in the reverse direction. The output of the algorithm is a representation of the similarity graph in edge-list format.

Unfortunately, the above algorithm suffers from severe performance degradation due to random *k*-tuple matches clogging *M *and slowing down list merges. *M *soon fills with many pairs containing low hit counts, representing sequences that are not significantly similar to *q *but share one or two *k*-tuples with *q *by chance. In fact it can be shown that under reasonable assumptions about the distribution of *k*-tuples in the database, the repeated list merging introduces an O(*D*^3^) factor into the running time.

SSAHA [29] overcomes the clogging problem by choosing *k *to be high enough that very few chance matches occur; however this is only a viable approach if enough memory is available as memory requirements are exponential in *k*. The impressive search speeds described in [29] were obtained on a computer with 16 Gb of RAM and with *k *set to 14 or 15. Requiring this amount of memory for coil would immediately put the program out of range of almost all computers in use today.

Another way to ameliorate the situation is to convert the list *M *into a form of *hashtable *by maintaining *r *separate pair lists *M*_0 _... *M*_*r*-1_, and merging the seqnum list for the *i*th *k*-tuple in *q *into the list *M*_*i*%*r*_. After all *k*-tuples have been scanned, a final merge step combines the *r *lists. Even better, partition by seqnum rather than *k*-tuple position: send each seqnum *i *to the list *M*_*i*%*r*_. The latter technique is more resilient to variations in seqnum list sizes. Choosing *r *= 2^*h *^for some positive integer *h *enables fast calculation of the remainder through bitwise operations. While these modifications do improve the running time of the naïve algorithm, as Table 1 shows, there remains much work to be done before this algorithm will be feasible for gigabyte-sized databases. We describe below a way to eliminate the time spent processing unpromising hits

**Table 1:** Execution time for find_edges variations on a small dataset

**Algorithm**	**Parameters**	**Execution Time (s)**
SSAHA	maxGap = 0,	118.58
	maxInsert = 0	
	maxGap = 20,	118.72
	maxInsert = 20	
Basic		97.98
Batch merging	*c *= 16	113.03
	*c *= 32	84.35
	*c *= 64	71.09
Recursive merging		80.55
Hashtable	*h *= 12	56.02
	*h *= 13	56.24
	*h *= 14	57.92

The dataset used, month.est_mouse, is a monthly update of the Genbank Mouse EST dataset comprising 31,401 sequences having average length 438 nucleotides.

### Letting go of perfection: the leaky move-to-front hashtable

The algorithms described in the preceding subsection all compute the complete list of (seqnum, hit count) pairs for a given query sequence *q*, including the "noise" matches with small hit counts, even though we end up keeping only the best *b *such matches. To avoid getting bogged down with noise matches, we modify the seqnum-hashing hashtable from the previous subsection by replacing each of the *r *variable-length linked lists in the hashtable with a small fixed-size array of size *f*. Instead of maintaining these arrays in seqnum order, a move-to-front discipline is used: whenever a seqnum *i *arrives, we scan the array *M*_*i*%*r *_for an occurrence. If it is found, it is moved to the front of the array, its hit count is incremented and all preceding elements are shunted down one position. If it is not found, it is inserted at the front of the array with hit count 1; all existing elements are shunted down one position to make room, with the last pair being deleted ("pushed off the end").

Intuitively, the success of this algorithm hinges on the following key assumption:

*If a database sequence is genuinely similar to the query sequence, its seqnum will turn up often enough that it will never be pushed off the end of the list*.

There are several reasons for the improved performance of this algorithm:

#### Frequently occurring seqnums are found more quickly and require fewer updates

A frequently occurring seqnum *x *is more likely to have been recently referenced and hence is more likely near the front of its array. Thus when *x *next occurs, the scan will not need to proceed very far down the array. Also note that only those elements that precede *x *in the array need to be shunted back – later elements remain in their original positions.

#### Packing the seqnum and count values

A "side effect" of the hashtable structure is that there is no need to explicitly record the low *h *bits of each seqnum in each array, since every seqnum in array *M*_*i *_must have these bits equal to *i*. Thus these bits are available for other uses. Since each array entry consists of a (seqnum, count) pair, and since count rarely exceeds *L*/*s*, it makes sense to store the count value in these bits. For our default choice of *k *and *s *parameters, this is reasonable for *h *≥ 10. (If it is important to deal properly with the rare occasions that that more 1023 *k*-tuples match between two sequences, we can simply saturate the count at 2^*h *^- 1.)

#### Fixed-size multidimensional arrays require very low memory overhead

The leaky move-to-front hashtable data structure is a 2^*h *^× *f *array. Because of their highly regular structure, accessing data items in fixed-size multidimensional arrays requires only multiplications and additions using the fixed dimension sizes, and does not require any pointers or special list termination symbols to be explicitly stored in memory, reducing the memory allocation overhead.

#### Computers like fixed-size arrays

Linked lists are efficient in theory, but in practice, computer hardware has long been designed for efficient processing of contiguous arrays of elements, and the "pointer-chasing" inherent in working with linked lists inevitably introduces comparatively large overheads. In particular, items at consecutive positions in a linked list may occupy widely separated memory addresses – a problem known as *poor spatial locality of reference*. In these circumstances, when iterating through the items in a linked list, memory cache hardware cannot predict which bytes will be read or written next, and performance suffers. In contrast, a scan through an array accesses memory bytes in sequential order, and will benefit from cache line fills that read contiguous blocks of memory into cache.

Assuming pessimistically that every seqnum must endure a full *f *comparison and move operations, analysis gives a time complexity of O(*fD*^2^/*s*4^*k*^) for this algorithm.

### Pentium 4 optimised version

Many modern CPUs use *pipelining *to increase instruction throughput. We have developed an implementation of the find_edges algorithm optimised for the heavily pipelined Pentium 4 processor [see Additional file 1].

### Extracting the encoding graph

Once a similarity graph has been created with find_edges, the next step is to extract from it an encoding graph that distinguishes groups of similar sequences and describes how they are to be encoded. Then each group is compressed independently. Both steps are performed by the program encode. For the time being, we assume the availability of a subroutine for delta-encoding one sequence in terms of another that produces a "black box" block of data bytes; this algorithm is described in the subsection "Delta-encoding Sequences".

First we note some structural properties of the encoding graph. Each sequence in the database will be either raw-encoded, or delta-encoded in terms of one other sequence: this implies that each vertex in the encoding graph will have at most one in-edge. Then by prohibiting cycles it is easy to show that the encoding graph will be a forest of directed trees, each having edges directed away from a root vertex. Since the decision about which vertex to choose as the root has little bearing on the speed or compression level achieved for a tree, coil selects the lowest-numbered sequence.

To be effective in compressing sequence databases, coil needs to produce an encoding graph in which highly similar sequences are linked by an edge whenever possible. More precisely, we want to maximise the total similarity score of the encoding graph, subject to the constraints that it be a subgraph of the similarity graph, and also a forest. This is the *maximum spanning forest *problem, which is equivalent to the *minimum spanning forest *problem using negative edge weights, which in turn is a generalisation of the heavily studied *minimum spanning tree *(MST) problem. Happily, several algorithms exist for efficiently solving these problems [22,31,32].

Since the similarity graph produced by find_edges is very sparse (containing at most *bN *edges) and is already in edge-list format, we employ Kruskal's O(|*E*|log |*E*|) algorithm [31]. Kruskal's algorithm is very simple to state:

1. Read in the similarity graph edge list.

2. Sort edges by similarity score.

3. For each edge in the sorted list:

• If this edge would not introduce a cycle, add it to the encoding graph forest.

Importantly, both sorting and cycle-testing can be performed efficiently. Edge sorting is accomplished in O(|*E*| + max(*score*)) time using bucket sort. (Note that the score of an edge between sequences of lengths *x *and *y *is at most min(*x*, *y*)/*s*). Cycle-testing is performed using the fast union/find data structure described in [33]. This data structure manages an equivalence relation on a set: here, the classes are the connected components of the encoding graph, which combine as edges are added. Determining whether an edge would induce a cycle amounts to testing whether its two endpoints are in the same class. *m *such tests can be performed on a set of size *n *in O(*m*α (*n*, *m*)) time [34], where α(*n*, *m*) is the extremely slow-growing inverse Ackermann function: effectively constant time per test.

### Delta-encoding sequences

Once the encoding graph has been created, encoding of the individual trees can begin. For each tree, the root sequence is output verbatim, and an in-order tree traversal then delta-encodes every other sequence in terms of its parent. It is well-known that such a traversal can be used to encode a rooted tree as a string containing only vertex identifiers and parentheses. Conversion in both directions can be accomplished without requiring random access to the characters of the string, implying that an in-memory tree data structure can be efficiently "streamed" to or from a sequential storage medium (such as a disk file) in time linear in the number of nodes.

An *edit script *is a list of edit operations, which we here take to be single-character insertions, deletions and substitutions. Our task is to find a minimal-length edit script for converting one string *a *of length *n *into another string *b *of length *m*. This problem can be solved in O(*nm*) time using a straightforward dynamic programming approach, in which we successively compute optimal edit scripts for pairs of prefixes of *a *and *b *in terms of previously computed solutions. Several algorithms exist that are asymptotically faster for certain input distributions [28,35]. In particular, an algorithm of Myers [35] can solve a variant of this problem in which only insertions and deletions are allowed in O(*nd*) time and space, where *d *is the edit distance (and thus the size of the edit script). Since the encoding stage of coil deals only with sequences already deemed to be similar by heuristics, this algorithm was chosen for implementation. Another attractive feature of the Myers algorithm is that it considers possible edit scripts in increasing order of edit distance, and can be terminated when the edit distance reaches some predetermined maximum distance *d*_max_. In coil's encoding stage, this is used to bound the runtime of the algorithm: if the edit distance between a pair of sequences exceeds a user-specified figure (defaulting to 150), the algorithm terminates early and a trivial edit script having length *a *+ *b *is produced.

Once an edit script has been found, it must be compactly encoded into data bytes. coil uses a simple scheme in which the most significant bit (MSB) of a byte specifies whether an insertion or deletion is to take place, and the remaining seven bits specify the offset (with respect to the source string) from the previous edit operation. If an edit operation is more than 126 characters along from the previous edit operation, a special code byte, having its lowest seven bits equal to 127, is emitted, indicating that the next byte should be read and 126 should be added to that byte's value to form the offset. This code byte may occur multiple times, adding 126 to the total offset each time. Since deletion operations identify character positions within the source string while insertion operations identify positions *between *characters, special care must be taken to handle string positions and offsets in a manner that permits unambiguous decoding.

In the case of an insertion operation, the character to be inserted is not recorded in-place but written to a separate file. This breaks the "edit script as black box" design principle, however separating the edit script and nucleotide data streams in this way makes the distributions of bytes in each stream more predictable, resulting in compression gains that cannot be overlooked.

Although the encoding described is fairly compact, it is clearly not optimal: for example, we expect position offsets to be tightly clustered around zero, implying that an encoding in which lower offset values were represented with fewer bits would yield higher space savings. However, this and any other detectable redundancy will be eliminated when the coil archive files are passed through an external general-purpose compression program.

### Sequence buffering

We have developed a simple buffering system that enables maximally efficient random access to sequence data [see Additional file 2].

### Incremental compression

Large sequence databases such as GenBank [36] are not static. They are being updated daily, and there is a need for database users to access the latest versions. The solution found by most organisations distributing these databases is to make available daily or weekly updates in the form of deltas – lists of sequences added, changed or removed from the original database release. End users who already have the main database installed can download the updates and apply these changes to their local database copies to produce up-to-date versions. These database deltas are much smaller files, often less than 100 Mb in size, and coil's usual mode of compression performs poorly on such small files.

A common update performed on a database is the addition of one or more new sequences. coil therefore supports *incremental compression*: the ability to efficiently encode one sequence database, the *increment*, in terms of another *baseline *database. We presume a user who downloads a database delta already has the original baseline database, so we can "refer back" to baseline sequences from within the encoding graph of the compressed increment. This approach makes available a large pool of candidate root sequences that can be used for efficient delta-encoding of each sequence in an increment.

In the remainder of this section we will refer to the mode of compression discussed in previous sections as *standalone *compression. For concreteness we will talk about compressing an increment database named incr.fasta in terms of a baseline database named base.fasta. It may be helpful to first read the subsection "Using coil", which describes the overall workflow and the individual files read and written by the various programs in the coil package.

### Implementation of incremental compression

Suppose that there are *B *sequences in the baseline database base.fasta, and *I *sequences in the increment incr.fasta. Compression of the baseline entails ordinary standalone compression; the only difference is that the *k*-tuple index files, and also the base.coil.seqnos file, need to be retained for compression (though not for decompression) of the increment. To compress the increment, *k*-tuple index files are produced from the increment database itself. However, we require the seqnums of the increment to be distinct from those of the baseline, so the -i *B *command-line option must be used with the make_index and find_edges programs to offset the starting seqnum by *B*.

The encode program is then run with the command-line option -i base, to indicate that the input file should be encoded in terms of the coil archive base. When this option is chosen, the Kruskal maximum spanning forest algorithm is modified to avoid adding an edge between two components which both contain baseline sequences. This is easy to accomplish, since all baseline sequences have seqnums less than *B*, and as components are identified by their lowest-numbered seqnum any component that contains a baseline sequence will be represented by the seqnum of that sequence, effectively limiting the involvement of baseline sequences to being the roots of components in the encoding graph.

Once encode has produced an encoding graph, it needs access to the sequence data so that delta-encoding can be performed. Obtaining the sequence data for an increment seqnum can be accomplished in the usual way (via the sequence buffering system), but this is not the case for baseline sequences.

The necessary baseline sequences are obtained from the baseline coil archive by decompressing the entire baseline database in-memory, but writing out only those baseline sequences which are the roots of trees in the increment encoding graph (which we call *buds*). During this step, baseline sequences will be visited in *decode order*; by first sorting the list of required baseline seqnums into this order, extraction can take the form of a list merge. The sorting step involves inverting the permutation of baseline seqnums recorded in the base.coil.seqnos file (a linear-time and -space operation), hence the requirement that this file be retained after compression of the baseline database. Then the encode program traverses and encodes all trees rooted in baseline sequences in decode order. For each such tree, the (strictly increasing) position of the baseline root sequence in the decode order is written to the file incr.bud, which is included in the increment archive to facilitate decompression. Finally, the program traverses and encodes all trees rooted in increment sequences in the usual fashion.

When decompressing an increment with the program decode, the command-line option -i base is used to specify that the coil archive base should be used as the baseline. decode first decompresses the baseline sequences at the positions listed in incr.bud and uses these sequences as roots for decoding the initial segment of the increment archive; then the remaining trees are decompressed as usual.

### Using coil

**Figure 3:**
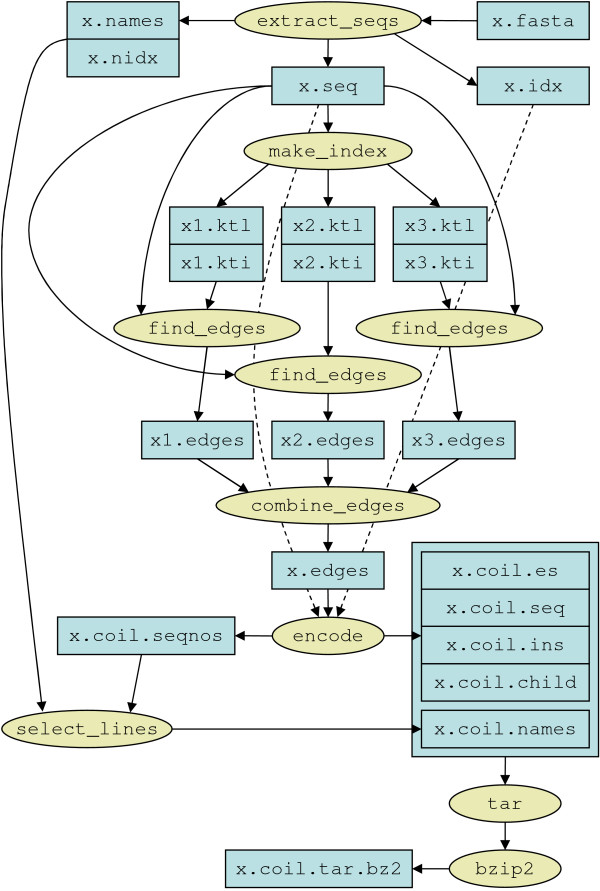
**Using coil to compress a FASTA database**. As few two-file *k*-tuple index segments are produced as memory allows.

Compressing a FASTA database using coil involves running several C programs that work together to produce a number of output files. Some of these files, collectively termed the coil *archive*, are required for recovering the original data, while the remainder may be discarded once compression is complete. Alternatively, the user may run a Perl script which automates these steps. The final step requires the files comprising the coil archive to be compressed by a general-purpose compression program. Figure 3 shows the complete process of using coil for standalone compression of a database; incremental compression is similar, but requires that all output files produced during baseline compression (including the baseline coil archive itself) are also available. Incremental compression produces one additional, small output file ending with the extension .bud which must be included in the final archive.

### Decompression

Decompressing a coil archive is simple: first "undo" the general-purpose compression used to compress the archive, then run the program decode. Decompression of an increment requires the name of the baseline database be specified on the command line with the -i switch. The process takes O(*D*) time and requires O(max(*seqLen*) * max(*treeDepth*)) memory. max(*treeDepth*) is typically small, but could be bounded using a simple adjustment to the Kruskal algorithm if necessary.

If the x.coil.seqnos file has been stored with the coil archive, then it is possible to recover the original sequence order at decompression time using the -x switch to the decode program; otherwise, sequences in the FASTA output file will appear in a different order.

For maximum portability across platforms, all files containing binary integer data use the little-endian storage format. All reading and writing of such files occurs through platform-specific load_vector() and save_vector() functions.

All programs accept the -h and --help switches, which can be used to display usage information. A brief description of each program can also be found in the README.txt file included in the coil software package.

## Results and discussion

To investigate the compression ratio achieved and running time required by coil for datasets of various sizes, tests were performed on randomly chosen subsets of sequences from a version of the GenBank est_mouse database, which contains 1,729,518,522 nucleotides in 3,852,398 sequences. Twelve dataset sizes were examined, with three test datasets produced for each size. Each dataset having a name of the form ems*n *contains 3,852,398 × *n*/100 sequences randomly selected from the est_mouse database. For the 100% size level, a single dataset (the original est_mouse database) was run three times, giving an indication of the noise level involved in execution time measurements.

A number of alternative compression programs were tested in addition to coil:

1. **bz2**: The general-purpose compressor bzip2 [27] with compression level 9.

2. **nrdb+bz2**: Elimination of duplicate sequences with the nrdb program, followed by bzip2 with compression level 9.

3. **PPMdi**: The PPMd general-purpose compressor variant I described in [37], with model order 8 and RAM usage 256 Mb (the most allowed by the program).

4. **7z**: the LZMA compression mode of the freely available general-purpose compression program 7-Zip [38]. This was the only other program we found that was capable of utilising 1 Gb of RAM during compression.

We also attempted to compress our datasets with the program DNACompress [9], however we found that we were unable to compress datasets larger than 14.5 million bases using this program. Unfortunately this is smaller than the smallest dataset we used in testing, and we were forced to abandon this attempt.

The tests on the ems*n *datasets specified restrictions for 7-Zip and coil to use at most 1 Gb of RAM. It should be noted that the other programs all use substantially less memory than this; in particular, PPMdi is limited to 256 Mb. To enable a fairer comparison with the PPMdi program, a further series of tests was carried out on the 140 Mb FASTA dataset rfam_full using 256 Mb settings for each program, and the -x option to coil.pl to enforce in-order sequence recovery. Due to problems attempting to compile nrdb on Windows, the nrdb+bz2 measurements were performed on a different computer: a Linux 3.2 GHz Xeon machine with 4 Gb of RAM. To measure decompression speed for nrdb+bz2, a simple C program, unnrdb, was written to expand the multi-header FASTA files produced by nrdb.

All coil runs used the parameter values *k *= 12, *s *= 8, *f *= 4 and *h *= 10, and were run on a 2 GHz Intel Core 2 Duo computer with 2 Gb RAM running Microsoft Windows XP. Most parameter values were chosen by earlier experimentation, however the choice of *f *and *h *received extra attention. Since it is important for the speed of the find_edges program that its hashtable data structure fit in cache memory, and it is not obvious how to trade off the *f *and *h *parameters for a fixed memory size, preliminary testing was conducted with several values of these parameters, suggesting (*f *= 4, *h *= 10) is best for the case where the hashtable is limited to 16 Kb in size – small enough to fit in the first-level cache of any modern computer system.

**Figure 4:**
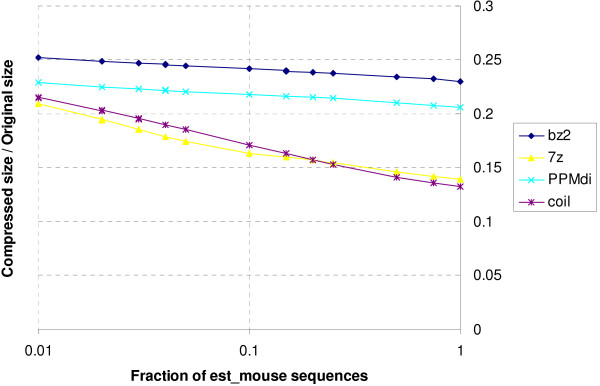
**Compression ratio vs. DB size**. The compression ratios of all tested algorithms increase as the input size increases; those of coil and 7-Zip increase faster than the rest.

Table 2 shows the sizes of the resulting compressed files. We immediately see that it is a race between coil and 7-Zip's LZMA compression mode: these two compressors easily outstrip all others, with the gap widening as file sizes increase. This is depicted graphically in figure 4. It appears that the 1 Gb of RAM available to both these compressors makes a big difference when compressing files containing many sparse repeats. There is never more than a 6% difference in file sizes between these two compressors, with 7-Zip performing best on smaller files. coil edges out 7-Zip on the larger files, eventually claiming a 5% improvement on the largest dataset tested, ems100.

**Table 2:** Compressed file sizes

**Dataset**	**FASTA**	**bz2**	**nrdb+bz2**	**7z**	**PPMdi**	**coil**
ems1	23292780	5876747	5871445	**4870989**	5331953	4990193
	23199910	5853780	5852865	**4854519**	5311350	4981279
	23201245	5852837	5852772	**4857631**	5312411	4988747
ems2	46519702	11576074	11574420	**9057588**	10475531	9432789
	46428669	11557030	11556573	**9023980**	10454826	9410376
	46390115	11547516	11549117	**9036246**	10445740	9426594
ems3	69631679	17211495	17205793	**12922145**	15537092	13607729
	69647486	17212318	17208461	**12907737**	15543489	13592739
	69715954	17231912	17225610	**12920294**	15558845	13623246
ems4	92905691	22841127	22810035	**16600091**	20601712	17625302
	93012024	22868732	22849091	**16611294**	20629724	17655369
	92850447	22813494	22799324	**16587471**	20585812	17584008
ems5	116125238	28428297	28415051	**20245345**	25636473	21509065
	116249077	28451622	28426520	**20260429**	25663621	21547174
	116117128	28413464	28397742	**20239745**	25630456	21496207
ems10	232365230	56136032	56054164	**37932764**	50662993	39774087
	232226017	56101887	56085818	**37910566**	50643774	39711435
	232230440	56099503	56030860	**37871106**	50622855	39685294
ems15	348404276	83539894	83461996	**55591757**	75411889	56758484
	348435883	83529794	83463158	**55594352**	75435650	56771053
	348292392	83453434	83396104	**55580937**	75374710	56768193
ems20	464825178	110838776	110755872	72989089	100113255	**72984372**
	464778933	110777795	110650470	**72991749**	100083039	73004561
	464532828	110766213	110653180	**72918789**	100046482	72978434
ems25	581105516	137940393	137814551	89636246	124600275	**88816000**
	580758935	137898843	137748733	89647136	124521398	**88829572**
	580693026	137884675	137756070	89594767	124526386	**88745435**
ems50	1161787240	272394718	271857439	169833915	244302747	**164139098**
	1161908810	272481687	271896055	169824808	244355206	**164069331**
	1161582289	272310746	271844248	169812165	244255108	**164093038**
ems75	1742471477	405262890	404293340	247835911	362403056	**236517596**
	1742664959	405243466	404268271	247921410	362419128	**236506851**
	1742458336	405281768	404397179	247684455	362394809	**236572552**
ems100	2323234744	533757352		324292321	478735224	**308211386**
	2323234744	533757352		324292321	478735224	**308211685**
	2323234744	533757352		324292321	478735224	**308211677**
ems100*	2323234744					308212275
rfam_full	140518668	4413613		4113889	9504648	**3995880**
	140518668	4413613		4113889	9504648	**3996447**
	140518668	4413613		4113889	9504648	**3995925**

All sizes are in bytes. The FASTA column shows the size of the original uncompressed FASTA file. The smallest file in each row is shown in bold. * This row shows the result of using version of find_edges optimised for the Pentium 4. nrdb+bz2 failed to compress the ems100 dataset because the size of the FASTA file exceeded 2 Gb. All coil runs performed on the rfam_full dataset used the -x option to enable in-order recovery of sequences. nrdb+bz2 was not used with the rfam_full dataset because it is incapable of restoring this order.

Looking at the execution times in Table 3, a similar transition takes place: coil is faster than 7-Zip up until around ems50, at which point the quadratic nature of find_edges starts to dominate. coil compresses the ems25 datasets faster and better than does 7-Zip by a small margin.

**Table 3:** Compression execution time

					**coil**				
					
**Dataset**	**bz2**	**nrdb+ bz2**	**7z**	**PPMdi**	find_edges	encode	tar+bz	other	**total**
ems1	5.6	7.7	43.3	5.6	3.5	9.3	1.2	10.8	24.8
	5.5	9.2	48.2	4.7	3.6	8.1	1.0	10.4	23.1
	5.7	10.2	43.5	4.3	3.6	8.2	1.2	12.5	25.5
ems2	10.3	15.1	96.5	9.9	9.5	20.0	1.0	19.1	49.5
	10.3	17.7	101.4	8.5	9.7	20.2	1.0	19.0	49.8
	10.1	16.8	95.6	8.3	9.7	20.3	1.1	20.8	51.9
ems3	15.2	22.6	154.8	14.7	17.0	36.5	1.9	27.7	83.1
	16.7	24.6	162.9	12.7	17.3	35.4	2.2	28.0	82.9
	15.4	22.4	154.2	12.8	17.3	34.5	3.1	29.2	84.2
ems4	20.3	32.0	216.5	20.0	25.8	50.6	3.6	34.3	114.3
	20.5	33.4	221.1	17.4	26.7	50.3	3.0	37.1	117.1
	20.0	31.1	215.0	17.0	26.0	49.1	3.3	39.3	117.7
ems5	30.3	39.1	276.7	25.3	35.5	65.4	4.2	42.7	147.7
	25.5	43.5	280.4	21.5	35.7	65.5	4.1	45.2	150.5
	25.3	38.7	275.9	21.4	35.8	64.6	4.1	46.2	150.7
ems10	62.5	85.1	573.8	49.8	100.5	179.3	8.4	100.4	388.6
	60.7	88.0	580.8	45.5	102.1	176.0	9.0	84.4	371.4
	50.6	80.1	575.4	43.5	100.5	160.8	9.4	87.7	358.4
ems15	94.3	117.8	871.1	69.0	197.9	271.8	12.6	118.2	600.6
	76.7	136.5	876.5	64.7	198.5	276.9	13.7	130.5	619.5
	89.8	119.6	869.5	64.5	196.1	275.8	13.8	133.4	619.1
ems20	101.5	169.9	1163.0	92.8	317.1	393.5	16.7	176.0	903.5
	101.7	179.7	1169.3	86.9	321.6	393.5	18.5	212.2	945.8
	120.5	158.8	1161.3	84.9	319.6	399.7	16.9	215.6	951.8
ems25	133.0	207.7	1482.2	116.0	471.7	503.2	22.5	280.8	1278.1
	152.3	220.7	1438.9	105.9	470.9	467.2	22.3	218.4	1178.8
	171.0	196.3	1456.4	106.0	468.0	504.4	23.4	248.0	1243.8
ems50	306.2	411.2	2882.0	215.4	1657.4	1172.3	105.4	716.3	3651.4
	340.0	452.4	2893.3	209.2	1658.2	1170.7	104.5	583.7	3517.1
	291.1	411.6	2888.3	207.2	1655.5	1174.7	107.9	671.8	3609.9
ems75	500.7	712.4	4328.8	314.4	3517.1	1814.9	167.8	1173.4	6673.2
	506.8	618.1	4304.5	311.9	3502.1	1810.2	164.7	992.9	6469.8
	508.7	593.5	4298.9	317.7	3490.7	1798.6	165.0	1116.4	6570.6
ems100	668.6		5760.8	408.5	6064.4	2552.4	223.9	1421.8	10262.6
	634.1		5707.5	404.1	6042.3	2524.8	219.3	1429.0	10215.3
	689.2		5773.6	403.3	6114.1	2496.4	217.5	1546.2	10374.1
ems100*					6446.3	2515.4	218.6	1505.8	10686.1
rfam_full	32.8		75.8	7.9	114.8	12.8	4.0	40.7	172.3
	29.6		75.3	7.9	113.9	12.3	4.3	38.2	168.7
	29.6		75.5	7.8	114.5	12.4	4.2	36.0	167.1

All durations are in seconds. The rightmost five columns break down the execution of coil by its main component programs; the "other" column includes the time needed for the programs extract_seqs, make_index and select_lines.

*This row shows the result of using the Pentium 4-optimised version of find_edges – surprisingly, this version of find_edges is actually about 6% slower than the original version on this CPU.

Finally, the decompression times shown in Table 4 show that coil is somewhat slower than the other programs, though still essentially linear-time as expected. Not shown in the table is coil's frugal memory usage during decompression – the maximum memory usage while decompressing ems100 is just 4.5 Mb, in comparison to the 89 Mb used by 7-Zip and the 270 Mb used by PPMdi.

**Table 4:** Decompression execution time

**Dataset**	**bz2**	**nrdb+bz2**	**7z**	**PPMdi**	**coil**
ems1	1.8	3.0	1.5	4.6	3.6
	1.8	3.1	1.5	3.9	4.5
	1.8	3.1	1.5	4.3	4.5
ems2	3.5	6.2	3.2	8.7	6.5
	3.5	6.1	3.2	7.9	6.6
	3.4	6.0	3.1	8.6	7.1
ems3	5.2	9.0	4.8	13.2	10.4
	5.2	9.3	4.9	11.7	11.0
	5.2	9.0	4.9	13.2	10.3
ems4	6.9	12.0	6.5	17.5	14.3
	7.0	12.3	6.4	15.6	13.6
	6.9	12.0	6.5	17.6	13.7
ems5	8.7	15.2	7.8	22.1	16.8
	8.6	15.3	7.3	19.7	17.7
	8.6	14.9	8.1	22.0	18.3
ems10	17.0	29.8	15.1	44.9	36.3
	17.0	30.6	14.8	40.1	36.8
	17.1	29.9	17.6	44.5	36.9
ems15	25.2	44.6	22.1	65.9	52.3
	25.5	46.0	22.0	59.1	53.7
	25.5	44.4	24.4	65.5	52.8
ems20	33.8	59.5	30.3	87.1	68.2
	34.0	60.7	29.8	78.1	70.2
	34.1	59.4	32.0	86.2	69.1
ems25	41.9	74.4	36.7	107.6	91.1
	42.1	76.3	38.9	100.9	85.9
	42.4	74.0	36.2	106.7	86.8
ems50	126.6	147.7	71.7	210.4	286.1
	128.6	152.0	71.9	202.8	274.9
	129.0	148.4	74.5	209.7	280.9
ems75	187.7	223.2	110.3	312.0	511.3
	190.5	228.3	111.3	306.7	471.3
	191.2	221.5	116.3	352.4	464.9
ems100	247.1		142.1	324.2	646.1
	248.6		137.6	404.9	674.2
	252.4		143.5	531.9	700.8
ems100*					649.9
rfam_full	9.6		7.9	9.1	60.7
	6.3		6.4	9.1	59.9
	6.1		6.2	9.1	60.2

All durations are in seconds. *This row shows the result of using the Pentium 4-optimised version of find_edges.

With respect to the rfam_full datasets, coil outperformed the nearest competition – again, 7-Zip – by around 3% in terms of compression ratio, though requiring more than twice as much time to do so. PPMdi performed poorly, producing a file more than twice the size of that produced by coil or 7-Zip. This is especially surprising given that these other programs were operating with the same 256 Mb RAM constraints as PPMdi for this dataset. bzip2 does substantially better with only 8 Mb of RAM at its disposal.

Surprisingly, although the optimised Pentium 4 version of find_edges produced a speed improvement of 25% on the Pentium 4 computer on which we performed initial testing, using this version of the program actually decreased performance by 6% on the Core 2 Duo platform. Only one test was run using this version of the program, indicated by a row with an asterisk in Tables 2, 3 and 4; all other results shown use the regular version of find_edges.

## Conclusion

We have demonstrated that the concept of edit-tree coding can be applied to produce a practical compression tool for sequence databases. The execution time required is not negligible and appears to grow quadratically with database size, but adequate compression on large EST databases can nevertheless be achieved on "everyday" modern computers. Furthermore, concern over compression time diminishes when it is considered to be amortised over the many decompressions that may take place in the targeted field of *one-source-many-sinks *operations. Decompression is acceptably fast, uses very little memory and can be performed on any computer with a C compiler. Source code portability and binary compatibility of compressed files has been tested on two widely used platforms, Linux and Win32.

There remains a wide scope for experimentation with coil and fine-tuning of algorithms and parameters. For example, one avenue not pursued here is the extent to which filtering of common *k*-tuples affects execution time and matching accuracy. It may be that the most commonly occurring 80% of *k*-tuples can be removed without dramatically affecting overall compression. While this kind of search space pruning would never be acceptable in a program like SSAHA that is specifically designed to find matches between sequences, we only care about accuracy of sequence matches where it noticeably improves compression.

## Availability and requirements

**Project name: **coil

**Project home page: **

**Operating system(s): **Tested on Windows and Linux. Binaries are additionally provided for Windows. Source code should compile in any UNIX-like environment.

**Programming languages: **ANSI C, Perl

**Other requirements: **Perl 5.6 or higher

**Licence: **BSD-style license

## Abbreviations

EST: Expressed Sequence Tag. In order to produce a protein, a cell first copies the segment of DNA encoding that protein (the *gene*) to a complementary *messenger RNA *molecule. ESTs are DNA sequences obtained by extracting and sequencing the messenger RNA molecules from a cell. Because only a single sequence read is performed on each molecule, ESTs are limited to approximately 800 bases in length. 

FASTA: A simple text file format for storing multiple DNA or protein sequences. Each sequence begins with a single line starting with the character ">" and containing the sequence name, followed by any number of lines containing the sequence data. 

MST: Minimum Spanning Tree. Given a connected, edge-weighted graph, a minimum spanning tree of the graph is a subgraph that (a) contains all vertices of the graph, (b) retains connectivity and (c) has minimum total weight among all such subgraphs. It follows that in a graph with positive edge weights, such a subgraph is always a tree.

## Authors' contributions

WTJW conceived the concept of edit-tree coding, designed and implemented the coil software, performed performance measurement, and produced an early draft of the manuscript. MDH provided design advice and made significant contributions to the final version of the manuscript.

## Supplementary Material

Additional file 1 Appendix 1 – Pentium IV optimised find_edges. Describes the version of the find_edges program optimised for the Pentium IV processor. Click here for file

Additional file 2 Appendix 2 – Sequence Buffering System. Describes the system used for efficiently obtaining random access to sequence data in memory-constrained environment. Click here for file
