# Supplementary material for: Compressing DNA sequence databases with coil
Source: BMC Bioinformatics. 2008 May 20;9:242. doi: 10.1186/1471-2105-9-242 (PMC2426707; doi:10.1186/1471-2105-9-242)
Supplement: Additional file 1 — Appendix 1 – Pentium IV optimised find_edges. Describes the version of the find_edges program optimised for the Pentium IV processor. [file 1471-2105-9-242-S1.doc]

# Appendix 1: Pentium IV Optimised find_edges

Many modern CPUs use *pipelining* to increase instruction throughput. In a pipelined CPU, each CPU instruction is fed through an execution pipeline consisting of several stages, so that typically each stage is processing a different instruction at any given time. The Intel Pentium 4 has 20 pipeline stages [27]. Performance is maximised when all pipeline stages are kept occupied with instructions, although this is not always possible: whenever one instruction’s behaviour depends on the outcome of an earlier instruction which has not yet completed executing, that instruction must “wait” for the earlier instruction to complete. In particular, a *conditional branch* instruction may change the memory address that the next instruction should be read from, depending on the outcome of some test (such as whether a particular CPU register is equal to zero) which is not yet known. Despite the existence of sophisticated branch prediction hardware in the Pentium 4, pipeline flushes due to mispredicted branches can dramatically reduce performance, especially when test outcomes are close to random.

Nearly all if … else constructs in compiled languages generate conditional branch instructions, so it follows that eliminating as many such constructs as possible from the inner loops of computationally-intensive code will improve the performance of that code on pipelined processors. Since the Pentium 4 is a very popular processor, we felt it was worthwhile to develop an optimised version of the central subroutine in the find_edges program for this processor. Our subroutine, which is written in assembly language, performs the step of merging a seqnum into a leaky move-to-front hashtable as described in the previous subsection, with the hardcoded restriction that *f* = 4. The code uses the SSE2 extended instruction set [28] to manipulate 128-bit quantities representing vectors of four 32-bit numbers. The fact that the code fits all relevant quantities into the eight SSE2 on-chip registers helps performance, however the most remarkable feature of this subroutine is that it manages to accomplish its task *without a single conditional branch instruction*. This is possible due to creative use of the PCMPEQD comparison instruction in concert with various bit-shifting and logical SSE2 instructions; for examples of the general approach the interested reader may refer to Chapter 2 of [29]. We feel that the 35 instructions of “straight-line” code we finally arrived at is close to the fastest possible implementation of this functionality on this processor. Performance results are described in the Results and Discussion section.
