# Supplementary material for: Compressing DNA sequence databases with coil
Source: BMC Bioinformatics. 2008 May 20;9:242. doi: 10.1186/1471-2105-9-242 (PMC2426707; doi:10.1186/1471-2105-9-242)
Supplement: Additional file 2 — Appendix 2 – Sequence Buffering System. Describes the system used for efficiently obtaining random access to sequence data in memory-constrained environment. [file 1471-2105-9-242-S2.doc]

# Appendix 2: Sequence Buffering System

The encode program must frequently deal with sequences which are widely separated in the input file, so it requires efficient random access to the sequence data. Although all POSIX-compatible operating systems provide an fseek() system call to allow a program to position the file pointer at any byte in a file, the subsequent read will almost certainly require the disk read/write head to physically move, a process which may consume several milliseconds. When performed several million times, this comparatively slow mechanical movement is inefficient.

This problem is addressed in coil by a buffering system which takes advantage of the fact that although random file seeks are slow, in-memory sorts are fast. Instead of performing delta-encoding during each in-order tree traversal, two passes through the entire forest are performed. Before the first pass, the sequence data file is scanned to extract an array of sequence file offsets, and a memory buffer of user-specified size (defaulting to 100Mb) is allocated. Then during the first pass through the encoding forest, tree traversals simply record the seqnums that need to be retrieved, adding each seqnum, along with its target offset in the memory buffer (computed using the file offset array), to the array ordered_seq_list. During the second pass through the encoding forest, the function get_next_seq() is repeatedly called to actually read the next sequence from the memory buffer and perform delta-encoding or raw-encoding as necessary. This function performs sequence buffering in a “lazy” fashion: if a sequence is available in the buffer, it will be returned; otherwise, the buffer_seqs() function is first called, which determines how many sequences will fit in the buffer, sorts this portion of ordered_seq_list by seqnum and reads the sequences into memory. Each sequence is placed in the memory buffer at the location determined by its position in the traversal order. The benefit of sorting the ordered_seq_list array is that sequences are read in file order, minimising disk seeks: in the limit case where the entire database fits in the buffer, no seeks at all are performed.
